# K13 Blocks KSHV Lytic Replication and Deregulates vIL6 and hIL6 Expression: A Model of Lytic Replication Induced Clonal Selection in Viral Oncogenesis

**DOI:** 10.1371/journal.pone.0001067

**Published:** 2007-10-24

**Authors:** Jinshun Zhao, Vasu Punj, Hittu Matta, Lucia Mazzacurati, Sandra Schamus, Yanqiang Yang, Tianbing Yang, Yan Hong, Preethello M. Chaudhary

**Affiliations:** 1 Division of Hematology-Oncology, Department of Medicine, Hillman Cancer Center, University of Pittsburgh, Pittsburgh, Pennsylvania, United States of America; 2 Spang Translational Research Core Facility, Hillman Cancer Center, University of Pittsburgh, Pittsburgh, Pennsylvania, United States of America; University of Hong Kong, China

## Abstract

**Background:**

Accumulating evidence suggests that dysregulated expression of lytic genes plays an important role in KSHV (Kaposi's sarcoma associated herpesvirus) tumorigenesis. However, the molecular events leading to the dysregulation of KSHV lytic gene expression program are incompletely understood.

**Methodology/Principal Findings:**

We have studied the effect of KSHV-encoded latent protein vFLIP K13, a potent activator of the NF-κB pathway, on lytic reactivation of the virus. We demonstrate that K13 antagonizes RTA, the KSHV lytic-regulator, and effectively blocks the expression of lytic proteins, production of infectious virions and death of the infected cells. Induction of lytic replication selects for clones with increased K13 expression and NF-κB activity, while siRNA-mediated silencing of K13 induces the expression of lytic genes. However, the suppressive effect of K13 on RTA-induced lytic genes is not uniform and it fails to block RTA-induced viral IL6 secretion and cooperates with RTA to enhance cellular IL-6 production, thereby dysregulating the lytic gene expression program.

**Conclusions/Significance:**

Our results support a model in which ongoing KSHV lytic replication selects for clones with progressively higher levels of K13 expression and NF-κB activity, which in turn drive KSHV tumorigenesis by not only directly stimulating cellular survival and proliferation, but also indirectly by dysregulating the viral lytic gene program and allowing non-lytic production of growth-promoting viral and cellular genes. Lytic Replication-Induced Clonal Selection (LyRICS) may represent a general mechanism in viral oncogenesis.

## Introduction

Kaposi's sarcoma-associated herpesvirus (KSHV), also known as Human Herpesvirus 8, has been etiologically linked to the development of Kaposi's sarcoma (KS), primary effusion lymphoma (PEL) and a subset of multicentric Castleman's disease (MCD) [1]–[3]. In infected cells, KSHV displays two distinct and alternative life-cycles: latent and lytic. Although herpesvirus oncogenesis has been generally attributed to the activity of latent proteins, lytic proteins are increasingly believed to play an important role in KSHV tumorigenesis [4]. However, since lytic replication eventually culminates in cell death, how the expression of lytic genes in cells destined to die can cause cancer has been a long-standing conundrum in the field. A possible solution to this problem was recently proposed and is based on the suggestion that dysregulated expression of lytic genes during latent phase or during aborted lytic cycles triggers KSHV tumorigenesis [5]–[7]. One such KSHV lytic gene that has been frequently implicated in the pathogenesis of KSHV-associated PEL and MCD, and may also have a role in KS development, is viral IL6 (vIL6), a structural and functional homolog of human IL6 (hIL6) [8]–[10]. Lytic replication of KSHV induces the expression of both vIL6 [11], [12] and hIL6 [13]. These cytokines act as B-cell growth and differentiation factors and have been shown to promote the survival and proliferation of KSHV-infected cells [14]–[17]. Additionally, they may contribute to the formation of bloody effusions, a characteristic feature of PEL, by stimulating angiogenesis and increasing vascular permeability by up-regulating the expression of vascular endothelial growth factor (VEGF) [17]–[21]. vIL6 may signal more promiscuously than hIL6 as it is not dependent on the gp80/IL6Rα-subunit of the IL6R complex and requires only the ubiquitously expressed gp130 receptor, whereas hIL6 requires both gp130 and IL6Rα for signal transduction [22], [23]. This property enables vIL6 to signal even in cells in which gp80/IL6Rα expression is down-regulated, such as those exposed to interferon-α, contributing to its additional role in immune evasion [16].

While the biological properties of vIL-6 described above are important to the pathogenesis of PEL, its unique expression pattern plays an equally important role. Although the KSHV genome is known to encode for homologs of several human chemokines and a G-protein coupled receptor (vGPCR) [4], the potential contribution of these proteins to the disease pathogenesis is limited by the fact that their expression is generally restricted to the lytic-phase of viral life-cycle and is observed in <1% of latently-infected PEL cells [24], [25]. In contrast, although vIL6 is a lytic protein, its expression is frequently detected in latently-infected PEL cells and in clinical samples of PEL, MCD and KS in the absence of other lytic genes, making it a particularly important cytokine in the pathogenesis of these diseases [8], [9], [24], [26]–[28]. However, despite the important role played by vIL6 in the pathogenesis of KSHV-associated malignancies, the molecular events leading to its dysregulated expression in latently-infected PEL, MCD and KS cells remain to be elucidated.

The open reading frame *K13* of KSHV encodes for a protein with two homologous copies of a death effector domain, which is also present in the prodomain of FLICE/caspase 8. A pivotal role for K13 in KSHV oncogenesis is supported by the facts that it is one of the few KSHV proteins to be expressed in latently-infected PEL and KS spindle cells [29], [30], and there is a dramatic up-regulation of its expression in late-stage KS, which is associated with a corresponding reduction in the rate of apoptosis in the lesions [29]. Based on its homology to caspase 8/FLICE, K13 was originally classified as a vFLIP (viral FLICE inhibitory protein) [31]. However, recent studies indicate that K13 does not act as an inhibitor of caspase 8 [32], [33]. Instead, it is now generally believed that K13 primarily acts as an activator of the NF-κB pathway [32]–[35], and utilizes this pathway to promote cellular survival, proliferation, transformation, and cytokine secretion [32], [36]–[40].

In this study, we have examined the role of K13-induced NF-κB on lytic reactivation of KSHV. We report that K13 differentially modulates the expression of KSHV lytic genes, resulting in not only enhanced survival of cells that were destined to die from lytic reactivation-induced cell death, but also dysregulated expression of viral and cellular IL6, which have been previously implicated in KSHV tumorigenesis.

## Results

### Generation of BCBL1-TREx-RTA-K13-ER^TAM^ cells

**Figure 1:**
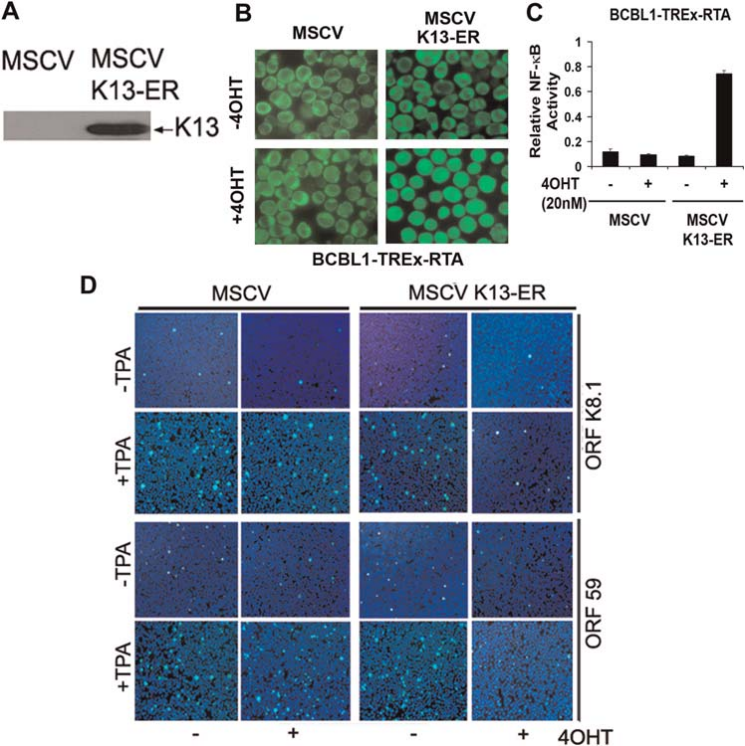
K13 blocks lytic replication in BCBL1-TREx-RTA cells. A. Expression of K13-ER^TAM^ in BCBL1-TREx-RTA cells as determined by immunoblotting with a Flag antibody. B–C. Treatment with 4OHT induces nuclear translocation (B) and DNA-binding (C) of p65 in BCBL1-TREx-RTA cells expressing K13-ER^TAM^ but is without effect in the control cells. Nuclear translocation was measured by indirect immunofluorescence analysis using a p65/RelA primary antibody (Santa Cruz Biotechnology). D. Inhibition of TPA-induced K8.1 and ORF59 expression by K13. BCBL1-TREx-RTA cells expressing an empty vector and K13-ER^TAM^, respectively, were left untreated or pretreated with 4OHT for 18 h and then induced with TPA for 96 h. K8.1 and ORF59 expression was detected by indirect immunofluorescence analysis with the indicated antibodies and revealed by Alexa-488-conjugated secondary antibodies. Nuclei were counterstained with Hoechst 33342. Cells were imaged with an Olympus Fluorescent microscope equipped with a SPOT camera. A representative of two independent experiments is shown.

PEL-derived BCBL1 cells are persistently infected with KSHV but can be induced to undergo lytic replication upon treatment with 12-*O*-tetradecanoyl-phorbol-13-acetate (TPA), which activates the expression of KSHV replication and transcription activator (RTA) [41]. A clone of BCBL1 cells, designated BCBL1-TREx-RTA, expresses RTA from a tetracycline-inducible promoter and undergoes a complete cycle of viral replication upon treatment with doxycycline [42]. BCBL1 cells express extremely low levels of endogenous K13 [35], [40], and demonstrate weak NF-κB activity as compared to the BC1 and BC3 PEL cell lines (Figure S1). In order to study the effect of K13 on KSHV lytic replication, we generated a polyclonal population of BCBL1-TREx-RTA cells with stable expression of a K13-ER^TAM^ fusion construct. The K13-ER^TAM^ construct expresses the K13 cDNA in-frame with the ligand-binding domain of a mutated estrogen receptor. The mutated estrogen receptor in this fusion construct does not bind to estrogen, its physiological ligand, but binds with very high affinity to the synthetic ligand 4OHT (4-hydroxytamoxifen) and regulates the activity of its fusion partner *in cis* in a 4OHT-dependent fashion [43], [44]. Thus, while the K13-ER^TAM^ fusion protein is expressed constitutively, it becomes active only on addition of 4OHT [43], [44]. We confirmed the expression of the K13-ER^TAM^ protein by immunoblotting (Figure 1A) and demonstrated tight regulation of its activity upon 4OHT treatment by immunofluorescence staining for p65/RelA subunit of NF-κB. As shown in Figure 1B, p65/RelA subunit was localized in the cytosolic compartment in both the empty vector- and K13-ER^TAM^ -expressing cells in the uninduced state, thereby demonstrating a lack of leakiness in the system. Treatment of K13-ER^TAM^ cells with 4OHT led to near uniform nuclear translocation of p65/RelA, and was accompanied by a corresponding increase in the NF-κB DNA binding activity, but had no effect in the control cells (Figures 1B and 1C). The NF-κB activity induced by 4OHT in K13-ER^TAM^ cells, however, was still within the physiological range as it was less than the endogenous NF-κB activity present in the BC1 cell line (Figure S1). Taken collectively, the above results demonstrate the suitability of the BCBL1-TREx-RTA-K13-ER^TAM^ cells for studying the effect of K13 on KSHV lytic replication.

### K13 blocks TPA-mediated lytic replication in BCBL1 cells

To study the effect of K13 on KSHV lytic replication, BCBL1-TREx-RTA-K13-ER^TAM^ cells were left untreated or treated with 4OHT for 12–18 h before treatment with TPA (20 ng/ml), and induction of lytic replication was examined by immunofluorescence staining for the early and late lytic proteins, ORF59 and K8.1, respectively. In the absence of prior treatment with 4OHT, ORF59 and K8.1 expression were detected in only a small fraction of cells, but was substantially increased upon treatment with TPA for 48–96 h (Figure 1D). Remarkably, pretreatment with 4OHT markedly reduced TPA-induced K8.1 and ORF59 expression in K13-ER^TAM^ cells (Figure 1D). 4OHT had no inhibitory effect on TPA-induced lytic gene-expression in the control vector (MSCV)-expressing cells, thus ruling out the possibility that 4OHT inhibits TPA-induced lytic gene expression independent of its effect on K13 activation (Figure 1D). Essentially similar results were obtained in K13-ER^TAM^ -expressing JSC-1 PEL cells (Figure S2), which are infected with both KSHV and the Epstein-Barr Virus (EBV), and possess low NF-κB activity in their basal state (Figure S1).

### K13 blocks RTA-induced lytic replication

**Figure 2:**
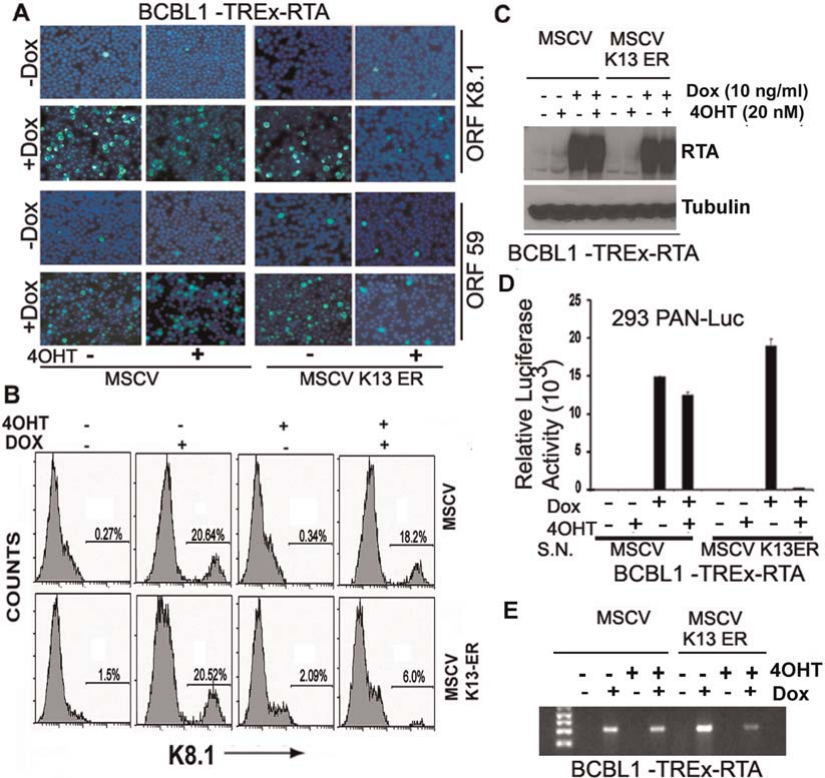
K13 blocks RTA-induced KSHV lytic reactivation. A. 4OHT treatment blocks RTA-induced K8.1 and ORF59 expression in K13-ER^TAM^ cells. The experiments were performed essentially as described for Figure 1D with the exception that RTA expression was induced by treatment with doxycycline (10 ng/ml). A representative of two independent experiments is shown. B. Flow cytometry analysis showing inhibition of RTA-induced K8.1 expression by 4OHT pretreatment in K13-ER^TAM^ cells. C. Equivalent induction of RTA upon doxycycline treatment in the vector- and K13-ER^TAM^ -expressing BCBL1-TREx-RTA cells in the absence or presence of prior treatment with 4OHT. Cells were treated with the indicated doses of doxycycline for 72 h prior to immunoblotting. D. K13 blocks RTA/doxycycline-induced production of infectious virions. 293PAN-Luc cells were infected in triplicate in a 24 well plate with 200 µl of cell-free supernatant collected from cells described in 2A. 72 h post-infection, luciferase activity was measured in cell lysates. The values (Mean±SEM) shown are from a representative of three independent experiments performed in triplicate. E. A semi-quantitative PCR assay showing inhibition of RTA/doxycycline-induced production of infectious virions by K13 in the cellular supernatants collected in 2D.

Doxycycline-induced expression of RTA in the BCBL1-TREx-RTA is sufficient to trigger KSHV lytic-replication [42]. Therefore, we next examined the ability of K13 to block doxycycline/RTA-induced lytic-replication in BCBL1-TREx-RTA-K13-ER^TAM^ cells. To test this hypothesis, empty vector- and K13-ER^TAM^ -expressing BCBL1- TREx-RTA cells were left untreated or treated with 4OHT and subsequently treated with doxycycline (10 ng/ml) to induce RTA expression. Remarkably, similar to the previous studies with TPA, prior treatment with 4OHT led to near complete inhibition of doxycycline-induced K8.1 and ORF59 expression in the K13-ER^TAM^ cells but was without effect in the control cells (Figure 2A). Inhibition of RTA-induced K8.1 expression by 4OHT-pretreatment in K13-ER^TAM^ cells was also confirmed using Flow cytometry (Figure 2B). Western blotting confirmed equivalent induction of RTA upon doxycycline treatment in the empty vector and K13-ER^TAM^ cells in the absence or presence of 4OHT (Figure 2C), thereby arguing against the possibility that the lack of expression of lytic proteins in K13-ER^TAM^ cells is due to the loss of doxycycline-induced RTA induction.

### K13 blocks the production of KSHV virions

KSHV genome encodes for a non-coding polyadenylated nuclear RNA (PAN), which is expressed briefly following KSHV infection [45]. A reporter cell line which expresses the β-galactosidase cDNA under the control of the PAN promoter has been previously shown to respond to infection with KSHV in a sensitive and quantitative manner that accurately assesses the amount of infectious KSHV present [46]. We generated a similar 293 reporter cell line, designated 293-PAN-Luc, in which the expression of the firefly luciferase gene is under the control of the PAN promoter, and used it to study the effect of K13 on the generation of infectious virions following treatment with TPA. As shown in Figure 2D, exposure of 293-PAN-Luc cells to the cell-free supernatant from doxycycline-treated BCBL1-TREx-RTA-K13-ER^TAM^ cells led to a significant increase in luciferase activity as compared to exposure to supernatant from non-doxycycline-treated cells suggesting the presence of infectious virus. However, the amount of infectious virus was significantly reduced in the supernatant derived from doxycycline-treated K13-ER^TAM^ cells that had been pretreated with 4OHT (Figure 2D). Treatment with 4OHT had no significant effect on doxycycline-induced production of infectious virions in the vector-expressing cells, thereby arguing against the possibility that the observed effect was due to an effect of 4OHT on cellular targets other than K13 (Figure 2D). Essentially similar results were obtained when the viral particles in the cellular supernatants were measured using a semi-quantitative PCR-based assay (Figure 2E). Finally, pretreatment with 4OHT also blocked virions production following treatment of K13-ER^TAM^ cells with TPA (data not shown). Taken collectively, these results demonstrate that K13-mediated inhibition of lytic gene-expression is accompanied by a block in the production of infectious virions.

### Role of the NF-κB pathway in the inhibition of lytic replication by K13

**Figure 3:**
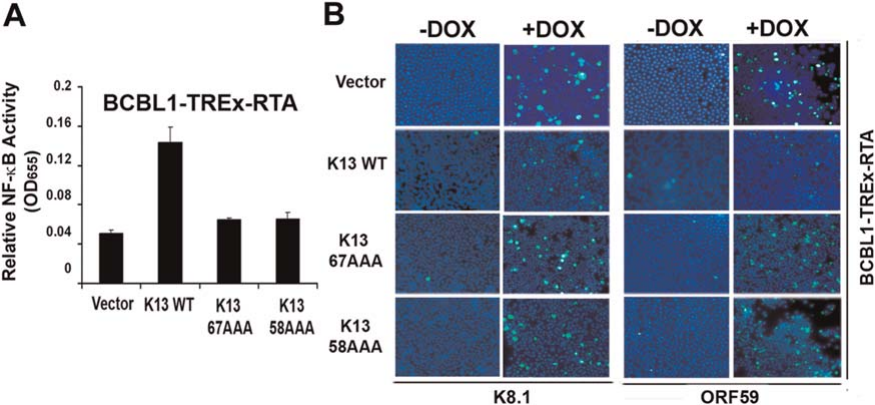
Role of the NF-κB pathway in the inhibition of KSHV lytic reactivation by K13. A. Status of the NF-κB pathway in BCBL1-TREx-RTA cells expressing wild-type K13 and its NF-κB-defective mutants, K13-58AAA and K13-67AAA, respectively, as measured by an ELISA-based DNA-binding assay. B. Wild-type K13 blocks doxycycline-induced K8.1 and ORF59 expression, while the NF-κB-defective mutants fail to do so. Cells were treated with doxycycline (10 ng/ml) and immunofluorescence analysis performed as described for Figure 1D.

To confirm the above results and to examine the role of the NF-κB pathway in the K13-induced inhibition of lytic replication, we generated stable clones of BCBL1-TREx-RTA cells expressing Flag-tagged wild-type K13 (i.e. without fusion with ER^TAM^) and its two NF-κB-defective mutants, K13-58AAA and K13-67AAA, respectively [37]. Constitutive expression of wild-type K13 in the BCBL1-TREx-RTA cells increased their NF-κB activity to a level approximately two-third of that seen in the BC1 cell line, while expression of K13-58AAA and K13-67AAA was without effect (Figure 3A and Figure S1). More importantly, while wild-type K13 effectively blocked doxycycline-induced K8.1 and ORF59 expression, no inhibition was observed in K13-58AAA and K13-67AAA-expressing cells (Figure 3B), thereby suggesting the involvement of the NF-κB pathway in the process.

### siRNA-mediated silencing of K13 expression induces lytic gene expression

**Figure 4:**
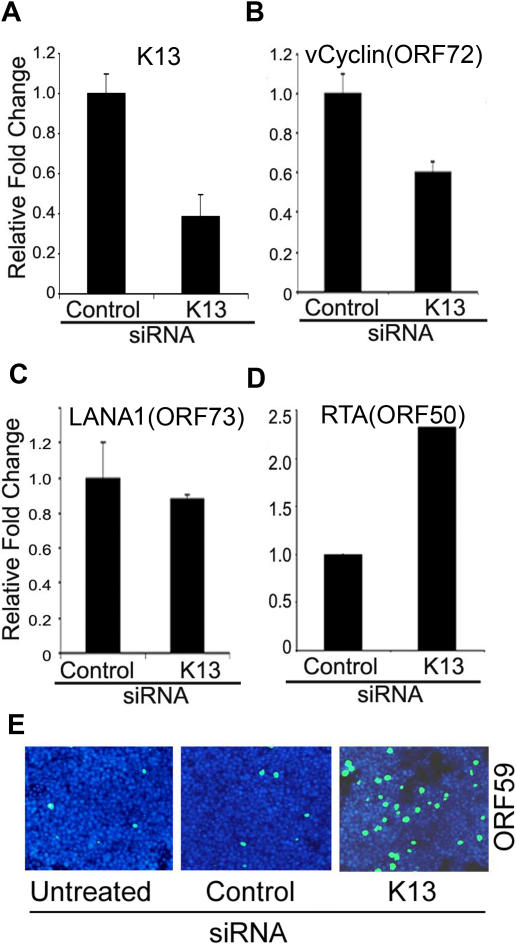
Down-regulation of K13 induces lytic gene expression. A–B. Cells were transfected with a control siRNA or a siRNA against K13 (Table S1) using oligofectamine (Invitrogen). Approximately 96 h post-transfection, down-regulation of K13 (A), vCyclin (B) and LANA-1 (C), and induction of RTA/ORF50 (D) gene expression was demonstrated by qRT-PCR. Real-time PCR reactions were performed in triplicate and the data presented as fold change in target gene expression (Mean±S.E.). E. Indirect immunofluorescence analysis showing up-regulation of ORF59 expression in cells transfected with K13 siRNA. Cells were analyzed 96 h post-transfection.

To rule out the possibility that the inhibition of lytic replication by K13 in the previous studies was an artifact of K13 over-expression, we examined whether silencing of endogenous K13 expression by RNA interference will induce lytic gene expression in PEL cells. For this purpose, BCBL1-TREx-RTA cells were transfected with a siRNA targeting K13 or an irrelevant control siRNA and specific down-regulation of K13 expression was confirmed by quantitative real-time RT-PCR analysis (qRT-PCR) (Figure 4A). Silencing of K13 by siRNA was accompanied by a parallel decrease in the level of vCyclin (Figure 4B), but had no significant effect on the level of LANA-1 transcript (Figure 4C). These results are consistent with previous reports and reflect the fact that the mature K13 transcript is present in the cells as part of a bicistronic transcript that also encodes vCyclin, whereas the LANA-1 coding region is structurally separated and present as a distinct transcript [40], [47]. More importantly, K13-silencing resulted in greater than two-fold induction of ORF50/RTA gene expression as determined by qRT-PCR (Figure 4D), and was accompanied by a significant increase in the expression of the lytic protein ORF59 (Figure 4E). These results support the hypothesis that endogenously expressed K13 promotes KSHV latency by blocking RTA expression and by blocking induction of RTA-responsive lytic genes.

### K13 protects cells against lytic replication-induced cell death

**Figure 5:**
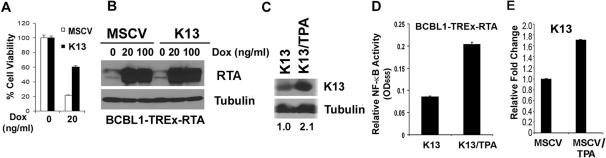
K13 protects cells against lytic replication-induced cell death. A. BCBL1-TREx-RTA-MSCV and K13 cells were treated with doxycycline (20 ng/ml) for 4 days to induce lytic replication. Cells were washed and incubated in doxycycline-free medium for 1 week. Cell viability was measured using MTS assay as described previously [36] and plotted relative to untreated cells. The values (Mean±S.E.) shown are from a representative of three independent experiments performed in triplicate. B. Equivalent induction of RTA upon doxycycline treatment in the vector- and K13-expressing BCBL1-TREx-RTA cells. Cells were treated with the indicated doses of doxycycline for 72 h prior to immunoblotting. C–D. Induction of lytic replication with TPA in BCBL1-TREx-RTA-K13 cells leads to the emergence of cells (K13/TPA) with increased K13 expression (C) and NF-κB activity (D), as measured by immunoblotting and an ELISA-based NF-κB DNA-binding assay, respectively. BCBL1-TREx-RTA-K13 cells were left untreated or treated with TPA (20 ng/ml) for 4 days followed by recovery in drug-free medium for 4 weeks prior to analyses. E. Induction of lytic replication in BCBL1-TREx-RTA-MSCV cells with TPA leads to emergence of cells with increase in endogenous K13 expression as measured by qRT-PCR. Treatment with TPA, followed by growth in drug-free media, was carried out essentially as in 4D. Real-time PCR reactions were performed in triplicate and the data presented as fold change in target gene expression (Mean±S.E.).

Since lytic replication of herpesviruses culminates in the death of the infected cell, we asked whether K13 might protect cells against lytic replication-induced cell death. Treatment of vector-expressing BCBL1-TREx-RTA cells with doxycycline led to a decline in cell viability, which was consistent with the induction of lytic replication (Figure 5A). However, doxycycline-induced decline in cell viability was substantially blocked in K13-expressing cells (Figure 5A). Western blotting confirmed equivalent induction of RTA protein in the vector- and K13-expressing cells, thereby arguing against the possibility that the increased survival of K13-expressing cells was due to their inability to induce RTA expression upon doxycycline treatment (Figure 5B). We also observed that K13-expressing BCBL1 and JSC-1 cells were more resistant to TPA- and sodium butyrate-induced cell death as compared to the control cells (data not shown). More importantly, the surviving clones obtained following the induction of lytic replication with TPA demonstrated significantly higher levels of K13 expression and NF-κB activity as compared to the untreated cells (Figures 5C–D). We also examined whether induction of lytic replication would also lead to the emergence of clones with an increase in the endogenous K13 expression. In the absence of an antibody capable of detecting the low level K13 present in the BCBL1 cells, we used real-time RT-PCR to measure endogenous K13 expression. As shown in Figure 5E, this assay revealed a significant increase in endogenous K13 expression in the surviving clones obtained following a single round of lytic replication with TPA. Thus, K13 confers survival advantage on KSHV-infected cells against lytic replication-induced cell death and, accordingly, induction of lytic replication selects for clones with increased K13 expression and NF-κB activity. These results might provide a possible mechanism for dramatic up-regulation of K13 expression observed in late-stage KS [29].

### K13 blocks TPA-induced up-regulation of RTA and RTA-target genes but fails to block vIL-6 induction

**Figure 6:**
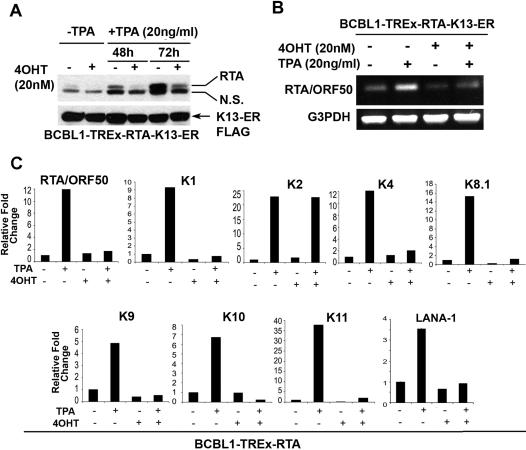
K13 differentially modulates the expression of KSHV genes following TPA treatment. A, B. 4OHT pretreatment effectively blocks TPA-induced RTA up-regulation in BCBL1-TREx-RTA-K13- K13-ER^TAM^ cells as measured by immunoblotting with an RTA polyclonal antibody (A) and RT-PCR analysis (B), respectively. G3PDH serves as a normalization control. C. Differential effect of K13 on the expression of TPA-target lytic genes and LANA-1. BCBL1-TREx-RTA-K13-ER^TAM^ cells were treated with TPA for 96 h with and without prior treatment with 4OHT and expression of the indicated genes measured by real-time RT-PCR analysis and normalized relative to GNB2L1 (housekeeping control). Real-time PCR reactions were performed in triplicate and the data presented as fold change in target gene expression (Mean±S.E.).

RTA is the master regulator of the switch between latency and lytic replication and TPA is believed to stimulate KSHV lytic replication by up-regulating RTA expression, which subsequently binds to and activates its own promoter in a positive feedback manner [48], [49]. Since NF-κB pathway is known to block the stimulatory effect of RTA on its own promoter and the promoter of KSHV lytic genes [50], we next examined the effect of K13 on the expression of RTA in the BCBL1-TREx-RTA-K13-ER^TAM^ cells. Treatment with TPA led to a time-dependent increase in RTA protein expression, which was significantly abolished by pretreatment with 4OHT (Figure 6A). A semi-quantitative RT-PCR analysis revealed that 4OHT blocks TPA-mediated induction of ORF50/RTA mRNA, suggesting that K13 blocks RTA expression at the level of gene transcription (Figure 6B). However, since K13 can also block doxycycline/RTA-induced lytic replication (Figures 2 and 3), inhibition of RTA expression may not in itself account for the inhibitory effect of K13 on lytic replication. Therefore, we next utilized real-time RT-PCR analysis to examine the effect of K13 on the expression of downstream RTA-target genes following treatment of BCBL1-TREx-RTA-K13-ER^TAM^ cells with TPA. As shown in Figure 6C, in addition to inhibiting induction of ORF50/RTA, 4OHT pretreatment significantly inhibited TPA-induced expression of several RTA target genes including K1, K4, K8.1, K9, K10, K11 and LANA-1. Taken collectively with the studies described in Figures 2 and 3, these results suggest that K13 not only blocks RTA expression but also blocks the transcriptional activation of RTA-target genes, resulting in the inhibition of lytic replication.

**Figure 7:**
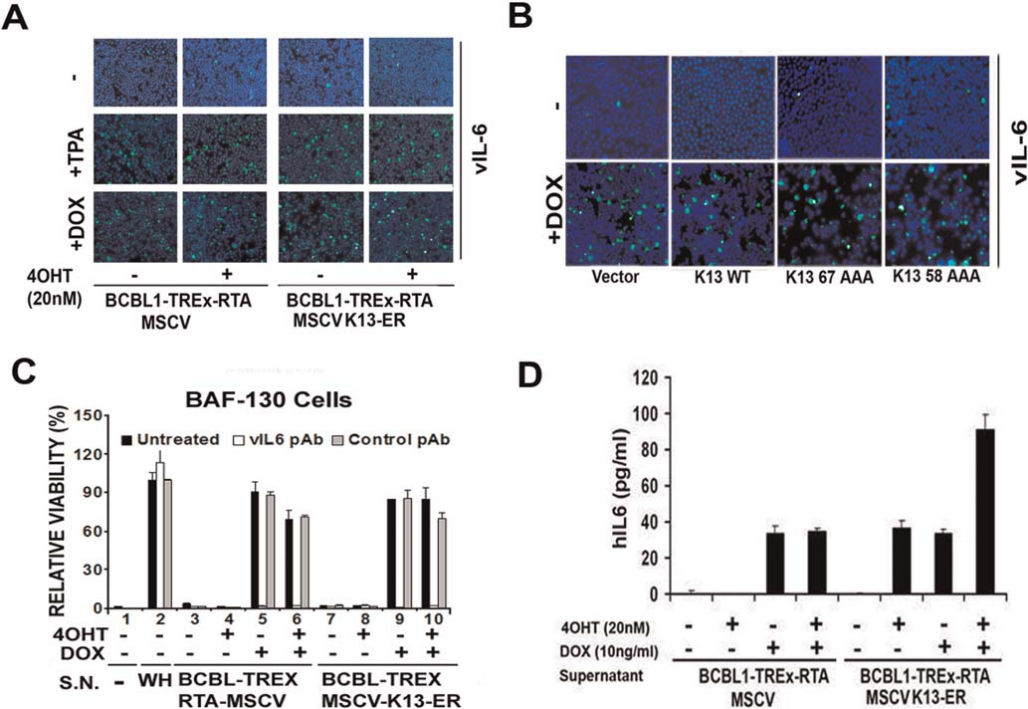
Effect of K13 on lytic replication-induced vIL6 and hIL-6. A. K13 activity fails to block TPA- and doxycycline-induced vIL6 induction. BCBL1-TREx-RTA-MSCV and K13-ER^TAM^ cells were treated with TPA (20 ng/ml) and doxycycline (10 ng/ml) for 96 h with and without prior treatment with 4OHT, followed by immunostaining with a vIL6 antibody. Nuclei were counterstained with Hoechst 33342. A representative of two independent experiments is shown. B. BCBL1-TREx-RTA cells expressing an empty vector, wild-type K13 or K13 mutants defective in NF-κB activation were treated with doxycycline for 96 h followed by immunofluorescence staining with an antibody against vIL6 as described in 7A. C. K13 fails to block RTA-induced vIL6 secretion. Culture of BAF-130 cells in the absence of WEHI-3B-conditioned medium (WH) led to a dramatic loss of cell viability (lane 1) which was rescued by the addition of supernatants (S.N.) from doxycycline-treated BCBL1-TREx-RTA-MSCV and K13-ER^TAM^ cells (lanes 5 and 9). Induction of K13 activity via 4OHT pretreatment had no inhibitory effect on vIL6 production (lane 10). Addition of a rabbit polyclonal antibody to vIL6 (300 ng/ml) effectively reversed the effect of doxycycline-treated supernatants on BAF-130 survival whereas a control antibody was without effect, thereby confirming the contribution of vIL6 to the observed effects. The values (Mean±S.E.) shown are from a representative of at least two independent experiments performed in triplicate. UT, untreated. D. K13 promotes RTA-induced hIL6 secretion. Secretion of hIL6 was measured using an ELISA kit (BD Biosciences, San Diego) in cell supernatants (10 µl) collected from BCBL1-TREx-RTA-MSCV and K13-ER^TAM^ cells that had been treated with doxycycline in the absence and presence of 4OHT, as described in Figure 7A. The values (Mean±S.E.) shown are from a representative of two independent experiments performed in triplicate.

While studying the effect of K13 on TPA-induced expression of RTA-target genes, we observed that it had no significant inhibitory effect on the induction of vIL6/K2 gene expression (Figure 6C). This was an intriguing result since vIL6 is not only an autocrine growth factor for KSHV-infected PEL cells, but also contributes to immune evasion and angiogenesis, and its dysregulated expression in latently infected cells has been implicated in the pathogenesis of both KS and KSHV-associated lymphoproliferative disorders [10], [16], [20]. Therefore, we carried out additional studies to confirm the results of RT-PCR analysis and to examine whether K13-induced inhibition of lytic replication in BCBL1-TREx-RTA-K13-ER^TAM^ cells is accompanied by dysregulated vIL6 expression at the protein level as well. Consistent with previous studies showing its increased expression during lytic replication, we observed significant vIL6 induction following treatment with either TPA or doxycycline (Figure 7A). However, in marked contrast to its effect on K8.1 and ORF59 expression (Figures 1 and 2), 4OHT had no significant inhibitory effect on TPA- or doxycycline-induced vIL6 induction (Figure 7A). Similarly, doxycycline resulted in equivalent induction of vIL6 in BCBL1-TREx-RTA cells expressing an empty vector, wild-type K13 or its NF-κB defective mutants (Figure 7B). The differential inhibitory effect of K13 on ORF59, K8.1 and vIL6 expression was also observed in the JSC-1 cell line (Figure S2).

While hIL6 requires both gp80 and gp130 signal transducers for cell signaling, vIL6 can engage gp130 independent of gp80 [22], [51]. To confirm the lack of a significant inhibitory effect of K13 on doxycycline-induced vIL6 production, we took advantage of gp80^−^/gp130^+^ BAF-130 cells [51]. These cells are derived from IL-3-depdendent Ba/F3 cells following transfection with a gp130 expression construct. The parental Ba/F3 cells lack the expression of both gp80 and gp130 (i.e. gp80^−^/gp130^−^) and therefore are unresponsive to both hIL6 and vIL6 [22]. Ectopic expression of the gp130 in the BAF-130 cells confers responsiveness to vIL6 while retaining insensitivity to hIL6, thus making them extremely useful for assaying the biological activity of vIL6 without interference from hIL6. Culture of BAF-130 cells in IL-3-free medium led to drastic loss of cell viability and proliferation (Figure 7C, lane 1), which were rescued by the addition of supernatants from doxycycline-treated BCBL1-TREx-RTA-MSCV or K13-ER^TAM^ cells (Figure 7C; lanes 5 & 9). Remarkably, supernatant from BCBL1-TREx-RTA-K13-ER^TAM^ cells that had been induced with doxycycline in the presence of 4OHT was equally effective in supporting the proliferation of BAF-130 cells (Figure 7C; lane 10). The contribution of vIL6 to the above protective effect was confirmed by the addition of a rabbit polyclonal vIL6 antiserum, which completely reversed the protective effect of the supernatants on BAF-130 viability and proliferation whereas a control rabbit antiserum was without effect (Figure 7C). Taken together, the above results demonstrate that K13-mediated inhibition of KSHV lytic-replication is not accompanied by a parallel block in the production of vIL6.

### K13 promotes RTA-induced hIL6 production

Similar to its viral counterpart, cellular IL6 (hIL6) has been also implicated in the pathogenesis of KSHV-associated lymphoproliferative disorders. Both K13 and RTA are known to induce hIL6 expression, albeit through different mechanisms [13], [39]. However, since K13 effectively blocks RTA-mediated up-regulation of K8.1 and ORF59 expression, we next examined whether it would also block RTA-induced hIL6 secretion. As shown in Figure 7D, induction of RTA expression with doxycycline led to increased hIL6 secretion in both BCBL1-TREx-RTA-MSCV and -K13-ER^TAM^ cells. Also, consistent with the published effect of K13 on hIL6 secretion, 4OHT treatment led to hIL6 induction in BCBL1-TREx-RTA-K13-ER^TAM^ cells, but was without effect in MSCV cells (Figure 7D). Importantly, contrary to its effect on RTA-induced K8.1 and ORF59 expression, 4OHT had an additive effect on RTA-induced hIL6 production (Figure 7D). Collectively, the above results demonstrate that while over-expression of K13 effectively blocks lytic replication-induced cell death by inhibiting the induction of RTA-target genes required for lytic replication, it has either a permissive or an additive effect on the production of growth-promoting viral and cellular cytokines, thereby contributing to the dysregulation of the lytic gene expression program observed during KSHV tumorigenesis.

## Discussion

### Role of K13 in the inhibition of KSHV Lytic Replication

Although originally classified as a vFLIP, K13 is now believed to be a potent activator of the NF-κB pathway and a key player in the pathogenesis of KSHV-associated malignancies [32]–[35]. In this report, we demonstrate that K13, one of the few KSHV-encoded latent proteins, blocks the switch from latent to lytic life-cycle by inhibiting the expression and transcriptional activity of RTA. Interestingly, similar to K13, LANA-1, which is another KSHV latent protein, can also inhibit lytic replication by blocking RTA expression and activity [52]. Thus, KSHV has devised multiple mechanisms to stringently control RTA expression and activity, thereby keeping lytic replication in check. Recent studies further suggest that KSHV-encoded vGPCR and K1 can also block lytic reactivation [53], [54]. However, unlike K13 and LANA-1, these proteins are expressed primarily during the lytic phase of KSHV life-cycle. Therefore, rather than preventing the onset of lytic replication, vGPCR and K1 may serve to slow its progression, thereby delaying cell death and allowing adequate time for virions assembly and release.

K13-induced NF-κB activation has been reported to play an essential role in promoting the survival of latently-infected PEL cells by up-regulating the expression of anti-apoptotic proteins [40]. Consistent with this role, siRNA-mediated silencing of K13 expression in PEL cell lines has been shown to result in the inhibition of constitutive NF-κB and induction of apoptosis [40], [47]. Our results showing induction of lytic genes following K13 silencing suggest that inhibition of lytic replication may also contribute to the pro-survival effect of K13 in PEL cells. Consistent with this notion, induction of apoptosis following siRNA-mediated silencing of K13 is a relatively delayed event, with peak apoptosis observed as late as 14 days post-siRNA transfection [40], [47], a time-course which is in accordance with the kinetics of cell death observed with lytic replication.

Over-expression of p65/RelA has been previously shown to block the stimulatory effect of RTA on lytic genes promoters, including its own promoter and the promoters of ORF57 and PAN genes [50]. Furthermore, treatment with Bay-11-7082, a specific inhibitor of the NF-κB pathway, is known to induce lytic replication in PEL cells [50]. Consistent with the above results, we demonstrate that the inhibitory effect of K13 on RTA expression and transcriptional activity is associated with NF-κB activation and is absent in K13 mutants that lack this activity. Taken collectively with the results of the previous study [50], our results support a role for NF-κB pathway in K13-mediated inhibition of KSHV lytic replication observed in the current study. However, it needs to be pointed out that there are five different NF-κB subunits that can combine as homodimers or heterodimers to affect a multitude of cellular genes and functions. Furthermore, the composition of NF-κB dimers and their function may vary depending on the cell type, the nature of the initiating stimulus and co-stimulation of other signaling pathways. Therefore, it is likely that the impact of NF-κB activation on KSHV lytic replication may not be straightforward and may depend, among other things, on the nature and magnitude of the NF-κB initiating stimulus and its timing with respect to the stimulus for lytic replication.

### Role of K13 in the Dysregulation of vIL6 Expression

Although latency is generally assumed to be the state leading to transformation by herpesviruses, proteins characteristic of viral lytic replication cycle have been regularly detected in KSHV-infected PEL, MCD, and KS cells, and implicated in tumorigenesis [4]. However, since the lytic genes are expressed in cells that are destined to die, this raises the question as to how lytic genes promote tumorigenesis. In the case of vIL-6, it has been observed that its expression is not restricted to the lytic phase, but can also be found in a significant fraction of latently-infected cells in PEL, KS and MCD in the absence of other lytic genes [8], [24], [26]. However, the underlying cause and the signaling mechanisms involved in the dysregulated expression of vIL6 in the latently-infected cells have not been clarified to date. In this report, we demonstrate that K13 is incapable of blocking RTA-induced vIL6 expression, which provides a possible explanation for the dysregulated expression of vIL6 in latently-infected PEL cells.

How does vIL6 escape from K13-induced inhibition? Although our study does not directly address this question, there are several nonexclusive possibilities. First, although RTA responsive elements (RRE) have been found in the promoters of several lytic genes, RTA does not recognize the same sequence element in all responsive promoters [55]–[57] and binds to different RREs with different affinities [58], which could account for their differential inhibition by K13. Second, RTA is known to activate its target genes through multiple mechanisms: direct DNA binding, protein-protein interaction with other cellular DNA-binding factors (e.g. RBP-Jκ), or both [55]. Differential involvement of transcriptional coactivators/repressors, including NF-κB subunits, on different lytic promoters might influence their response to K13. Finally, it is possible that transcriptional activation of different RTA target genes require different levels of RTA, so that even a small amount of transcriptionally active RTA present in K13-expressing cells may be sufficient to induce vIL6 expression while failing to induce the expression of other lytic genes. It needs to be clarified, however, that in the absence of experimental evidence in support of the above possibilities, they should be considered speculative at the present and their formal proof awaits further studies.

### A Model of Lytic Replication-Induced Clonal Selection (LyRICS) in Viral Oncogenesis

**Figure 8:**
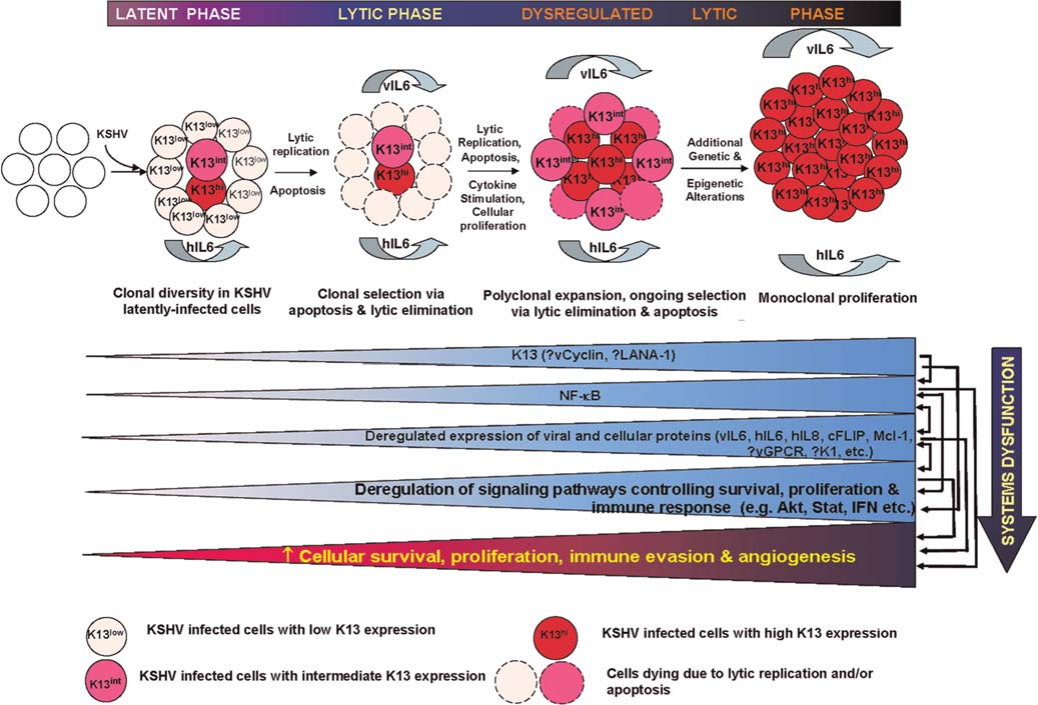
A speculative model of Lytic Replication-Induced Clonal Selection (LyRICS) in KSHV tumorigenesis. Infection with KSHV leads to a population of latently-infected cells with varying levels of K13 expression. Cells with low K13 expression are eliminated by apoptosis and lytic replication, while those with intermediate to high expression are stimulated to proliferate through the stimulatory effect of the NF-κB pathway on cell cycle and through the secretion of growth-promoting cytokines (e.g. hIL6). Elevated K13 expression also blocks the production of key viral proteins needed for lytic replication, thereby protecting cells from lytic replication-induced cell death. However, K13 has a permissive effect on RTA-induced vIL6 production and cooperates with it to stimulate hIL6 secretion further, thereby dysregulating the lytic gene expression program. The dysregulated expression of viral and cellular genes leads to further deregulation of signaling pathways controlling cellular survival, proliferation, immune response, and angiogenesis, initially leading to polyclonal expansion and, subsequently, through the accumulation of additional genetic and epigenetic alterations, to monoclonal cellular proliferation. Increased K13 expression, either alone or in combination with other viral (e.g. vCyclin, LANA-1, vGPCR and K1) and cellular proteins (e.g. proinflammatory cytokines) may lead to dysregulated expression of additional viral and cellular proteins in a cell-type and context-dependent manner, which may contribute to the pathogenesis of different cancers associated with KSHV infection. It is conceivable that acquisition of secondary genetic and epigenetic changes in the later stages of the disease may reduce or obviate the need for continuous K13 expression and NF-κB activity in some cases (not depicted).

In addition to providing a possible mechanistic explanation for the dysregulation of vIL6 expression, our results may have broader implications for the role of lytic replication in KSHV tumorigenesis. Thus, K13 is known to promote the survival of KSHV-infected cells [40], [47], protect against growth factor-withdrawal-induced apoptosis [36], and stimulate cellular proliferation and cytokine production [38], [39], [44], [59]. In the current study, we further demonstrate that K13 confers protection against cell-death induced by lytic replication. Taken collectively, these results raise the intriguing, though speculative, possibility that the low-level lytic replication, as is frequently observed in PEL, working in conjunction with increased cellular proliferation and protection against apoptosis conferred by K13 expression, may favor the emergence of clones with elevated K13 expression (Figure 8). In turn, elevated K13 expression and NF-κB activity may not only protect cells against future cycles of lytic replication, but further dysregulate the viral gene expression program, resulting in the non-lytic expression of vIL6 and enhanced hIL6 production (Figure 8). The resultant increase in proliferation, angiogenesis and immune-evasion, combined with inhibition of apoptosis, may lead to polyclonal expansion of cells with dysregulated viral and cellular gene expression programs, and following acquisition of additional genetic and epigenetic abnormalities, to the outgrowth of fully transformed clones (Figure 8).

It is important to note that while K13 is primarily responsible for constitutive NF-κB activation in PEL cells, additional mechanisms do exist for the activation of this pathway in KSHV-infected cells, including viral-encoded proteins capable of NF-κB activation, such as vGPCR and K1, both of which have been shown to block KSHV lytic replication [53], [54]. Interestingly, NF-κB activation is also a property shared by several inflammatory cytokines implicated in KSHV tumorigenesis [60]. Finally, NF-κB up-regulation in KSHV-infected cells may also result from over-expression and/or mutations of cellular proteins belonging to the NF-κB signaling pathway. Thus, it is conceivable that additional viral and cellular proteins cooperate with K13 in the activation of the NF-κB pathway, inhibition of lytic replication and dysregulation of viral and cellular gene expression programs during KSHV tumorigenesis (Figure 8). Additionally, since K13 mRNA is expressed as part of a bi- or tricistronic message with vCyclin and/or LANA-1 [61], selection of K13-over-expressing clones may simultaneously select for clones with elevated vCyclin and/or LANA-1, which may cooperate with K13 in causing progressive dysregulation of viral and cellular gene expression programs during KSHV tumorigenesis (Figure 8).

Lytic replication has been increasingly recognized as a key player in the pathogenesis of KSHV-associated malignancies [62]. However, most of the discussion of this topic has so far focused on its role as a source of new virions needed to recruit new cells to latency to replace those that have died or have lost the viral genomes, and as a source of lytic genes with growth-promoting and transforming abilities [4], [5], [7], [62], [63]. A novel aspect of our model is the recognition that lytic replication may also act as a selective force that, when operative over protracted time periods in the case of chronic infections, has the potential of driving the emergence and evolution of clones with progressive dysregulation of viral and cellular genes, with cancer being the final inadvertent outcome of this progressive systems dysfunction. Thus, rather than simply serving as a source of new virions or potential oncogenes, the process of lytic replication, in itself, may be inherently tumorigenic.

Several aspects of this speculative model may need further clarification. *First,* it is important to emphasize that our model does not exclude other roles of lytic replication in KSHV tumorigenesis that have been previously described in the literature [25], [62] and were briefly discussed above. On the other hand, we favor the hypothesis that KSHV tumorigenesis is the culmination of multiple complex interactions between the virus and its host, and lytic replication as a driver of clonal evolution and systems dysfunction, as outlined in our model, constitutes but one aspect of this dynamic and complex process. In fact, the full expression of the tumorigenic potential inherent in lytic replication may be influenced by a number of host and viral factors, such as the infected cell type, the host immune response, the degree and duration of lytic replication, and the nature, number and oncogenic potential of the dysregulated viral and cellular genes. *Second,* it is important to note that, due to the lack of a suitable model for studying KSHV lytic replication in endothelial cells, our model is based on experiments performed in PEL cell lines and, as such, it has primary relevance for KSHV-associated lymphoproliferative disorders. Nonetheless, since latently-infected KS spindle cells not only express K13 but also show its elevated expression with tumor progression [29], it is conceivable that K13 also contributes to the dysregulation of lytic gene expression program during KS pathogenesis. A potential criticism of our model when applied to KS, however, is the lack of evidence for aggressive lytic infection in early-stage KS that would justify lytic replication as the tumor driver. On the other hand, the process of lytic replication-induced clonal selection, as envisioned in our model, may manifest itself over several cellular generations. Therefore, from an evolutionary perspective, it is conceivable that even low-level lytic replication observed in the early-stage KS lesions may exert significant cumulative selective pressure to drive the emergence of clones with elevated K13 expression and dysregulated expression of viral and cellular genes. Another unique property of KS—their relatively low proliferative rate [64]—may also magnify the impact of even low-level lytic replication on tumor composition over time. Finally, although the level of lytic replication in early-stage KS is low, these lesions do demonstrate large numbers of apoptotic cell, and there is a dramatic decline in the number of apoptotic cells with lesion progression with an associated increase in K13 expression [29]. Therefore, it is conceivable that lytic replication acts in conjunction with apoptosis to drive the selection of clones with increased K13 expression, enhanced NF-κB activity, and dysregulated expression of viral and cellular genes.

It is important to clarify that the level of cellular K13 (or NF-κB) is not the sole determinant of lytic induction. Instead, the switch form latency to lytic is probably regulated by the expression and activity of a number of cellular and viral proteins and signaling pathways, and K13 is but one component of this cellular rheostat. Furthermore, increased K13 expression and NF-κB activity may play a dominant role during the early and middle stages of KSHV tumorigenesis, when there is need for exuberant cytokine production to promote the survival of virally-infected cells and to drive polyclonal expansion. It is conceivable that acquisition of secondary genetic and epigenetic abnormalities by rapidly proliferating cells may select for clones with the ability to grow in a cytokine-independent fashion. This may reduce, or obviate altogether, the need for continuous elevated K13 expression and NF-κB activity near the terminal-phase of the disease, which may provide an explanation for the relatively low K13 expression and NF-κB activity in some PEL cell lines, such as the BCBL1 cells used in the current study. Additionally, elevated K13 expression and NF-κB activity during tumor growth may be maintained under the constant selective pressure exerted by ongoing apoptosis and/or lytic replication, while the tumor is growing under harsh environmental conditions *in vivo*. As these selective forces may no longer operate on PEL cell lines growing under standard tissue culture conditions *in vitro*, this may result in a gradual loss of K13 expression and NF-κB activity over time, providing yet another explanation for the relatively low K13 expression and NF-κB activity observed in some PEL cell lines.

One of the great puzzles of KSHV oncogenesis has been the relatively low incidence of KS and PEL among immunocompetent individuals even in areas where the rates of KSHV seropositivity are relatively high, and the sharp increase in the incidence of these diseases upon introduction of immunosuppression due to HIV/AIDS or solid organ transplantation [65]–[67]. Since KSHV lytic replication is markedly enhanced in immunosuppressed patients [68]–[70], it is possible that the increased incidence of PEL and KS in patients with HIV/AIDS and transplant recipients may be in part due to the increased selective pressure exerted by ongoing lytic replication and the resultant accelerated emergence of clones with dysregulated viral and/or cellular gene expression programs. Interestingly, immunosuppression due to AIDS, organ transplantation, and acute malaria infection is also known to lead to increased lytic replication of EBV, and has been linked to increased incidence of EBV-associated malignancies [71], [72]. Thus, lytic replication-induced selection of clones with up-regulated survival signaling pathways and resultant dysregulation of viral and/or cellular gene expression programs may also contribute to the pathogenesis of EBV-associated malignancies, and possibly to other malignancies linked to viral infections. Interestingly, an important implication of the notion that lytic replication may promote tumorigenesis by acting as a selective force rather than as a source of new virions or virally-encoded oncogenes is that viruses may have a role in promoting even those cancers in which viral genomes have not been detected in the cancer cells.

Finally, while the association of ongoing lytic replication with increased incidence of cancer has been best studied in the context of KSHV tumorigenesis, increased cell death is a feature commonly seen in the early stages of most human cancers, including those associated with chronic infection, inflammation, exposure to environmental carcinogens, activation of oncogenes and loss of tumor suppressor genes [73], [74]. Thus, the paradigm of lytic replication-induced selection of clones with dysregulated survival signaling pathways proposed in this study may not be limited to viral carcinogenesis and may represent but one special case of a more general phenomenon of cell death-induced evolution of clones with systems dysfunction during cancer development. Indeed, similar to the role played by natural selection during evolution, excessive cell death, rather than its absence, may be the selective force driving clonal evolution during the initial stages of most cancers. This view of the origin of cancer, that we refer to as a Phoenix Paradigm, has obvious implications for not only a better understanding of cancer pathogenesis, but also for the development of effective strategies for its prevention and treatment, and deserves experimental confirmation.

## Materials and Methods

### Cell lines and constructs

BCBL1- TREx-RTA and JSC-1 cells were kindly provided by Drs. Jung (Harvard Medical School) and Richard Ambinder (Johns Hopkins University), respectively and were obtained from Dr. Frank Jenkins. BAF-130 cells were obtained from Dr. John Nicholas (Johns Hopkins University) with the kind permission of Dr. Kishimoto, and were grown in RPMI 1640 supplemented with 10% FCS and 10% conditioned medium from WEHI-3B cells as a source of murine IL-3. MSCVneo-based retroviral vectors expressing Flag tagged K13-ER^TAM^, wild-type K13 and its mutants have been described previously [37], [44] and were used to generate polyclonal populations of infected cells after selection with G418.

### Assay for infectious virions

A luciferase reporter construct containing the PAN promoter region spanning bp −122 to +14 was cloned in the pGL3 basic vector (Promega, Madison, WI) and transfected into 293 cells along with a plasmid conferring G418 resistance. Several independent clones were selected in G418 and a clone with low basal and a dose-dependent increase in luciferase activity upon infection with KSHV was selected for further analysis, and designated 293PAN-Luc. To analyze the presence of infectious virions in the supernatant of PEL cells, approximately 0.75×10^5^ 293PAN-Luc cells were plated in each well of a 24 well plate and next day infected in triplicate with 200 µl of cell-free supernatants collected from TPA or doxycycline-induced cells. Infection was carried out in the presence of polybrene (8 µg/ml), essentially as described [46]. Cells were lysed 48–72 h post-infection and lysates used for the measurement of luciferase activity as described previously [34]. Measurement of infectious virus in the cellular supernatants using a PCR based assay was done essentially as described previously [52] except that viral DNA was purified by an additional step of phenol-chloroform extraction followed by ethanol precipitation prior to PCR amplification. PCR amplification was carried out for 25 cycles using primers specific for a KSHV region located between ORF18 and ORF19 [52].

#### Induction of lytic replication and immunofluorescence analysis

In experiments involving K13-ER^TAM^ cells, pretreatment with 4OHT (20 nM) was carried out for 12–18 h. Cells were treated with TPA (20 ng/ml) or doxycycline (10–20 ng/ml) for 72–96 h to induce lytic replication following which supernatants were harvested for the measurement of infectious virions and cytokines, and cells fixed and used for indirect immunofluorescence analysis using an Olympus Fluorescent microscope equipped with a SPOT camera, essentially as described previously [44], or analyzed by Flow cytometry. For experiments involving siRNA-mediated silencing, cells were transfected with a control siRNA or a siRNA against K13 (Table S1) using oligofectamine (Invitrogen; Carlsbad, CA). Primary antibodies against K8.1, ORF59 and vIL6 were purchased from ABI (Columbia, MD) and revealed with Alexa-488 conjugated secondary antibodies (Invitrogen). Nuclei were counterstained with Hoechst 33342.

#### NF-κB assay

DNA binding activity of the p65/RelA NF-κB subunit was measured in triplicate in the nuclear extracts using the ELISA-based TransFactor kit (Clontech) following the manufacturer's recommendations.

### RT-PCR and Real-time PCR (qRT-PCR)

RNA was isolated using the RNeasy Mini kit (Qiagen) and semi-quantitative RT-PCR performed as described previously [44]. Real-time PCR reactions were performed in triplicate using an ABI Prism 7000 system and SYBR green-*Taq* polymerase mix to determine the relative change in the expression of various KSHV genes. GNB2L1 (Guanine nucleotide binding protein, beta polypeptide 2-like 1) was used as a housekeeping control. qRT-PCR data (Ct values) was analyzed using the 2^−ΔΔ C^_T_ method [75], and the data presented as fold change in target gene expression±standard error of mean. Primers used for real-time PCR are shown in Table S2.

## Supporting Information

Figure S1 Relative level of NF-κB activity and K13 expression in PEL cells. A. Basal level of NF-κB activity in different PEL cell lines as measured by the TransFactor ELISA-based assay kit. B. A NF-κB DNA binding assay showing the NF-κB activity present in BCBL1-TREx-RTA cells expressing a control vector (MSCV), K13 or the K13-ERTAM construct (with and without 4OHT treatment) as compared to the basal level of NF-κB activity present in the BC-1 cell line. DNA binding of p65 NF-κB subunit was measured using the TransFactor ELISA-based assay (Clontech). C. A qRT-PCR assay showing the relative level of K13 expression in the BCBL1-TREx-RTA cells expressing a control vector (MSCV) or K13 as compared to the basal level of K13 expressed in the BC-1 cell line. The qRT-PCR analysis was performed in triplicate and GNB2L1was used as a normalizing control.
(0.14 MB PDF)

Figure S2 K13 blocks lytic replication in JSC-1 cells. A. Expression of K13-ER^TAM^ in BCBL1-TREx-RTA cells as determined by immunoblotting with a Flag antibody. B. Treatment with 4-OHT induces NF-κB DNA-binding in JSC-1 cells expressing the K13-ER^TAM^ fusion protein. DNA binding of p65 NF-κB subunit was measured using the TransFactor ELISA-based assay (Clontech). C. K13 blocks TPA-induced ORF59 expression but fails to block vIL6 induction in JSC-1 cells. JSC-1-K13-ER^TAM^ cells were left untreated or treated with 4OHT (20 nM) for 24 h and then induced with TPA (20 ng/ml) for 96 h. Expression of ORF59 and vIL6 was detected by indirect immunofluorescence analysis. Nuclei were counterstained with Hoechst 33342.
(0.72 MB PDF)

Table S1 Sequence of siRNA oligonucleotides.
(0.01 MB PDF)

Table S2 Sequence of primers used for RT-PCR and qRT-PCR analyses.
(0.01 MB PDF)
