# Supplementary material for: K13 Blocks KSHV Lytic Replication and Deregulates vIL6 and hIL6 Expression: A Model of Lytic Replication Induced Clonal Selection in Viral Oncogenesis
Source: PLoS One. 2007 Oct 24;2(10):e1067. doi: 10.1371/journal.pone.0001067 (PMC2020437; doi:10.1371/journal.pone.0001067)

Figure S1. Relative level of NF-κB activity and K13 expression in PEL cells.

A. Basal level of NF-κB activity in different PEL cell lines as measured by the TransFactor ELISA-based assay kit.

B. A NF-κB DNA binding assay showing the NF-κB activity present in BCBL1-TREx-RTA cells expressing a control vector (MSCV), K13 or the K13-ER^{TAM} construct (with and without 4OHT treatment) as compared to the basal level of NF-κB activity present in the BC-1 cell line. DNA binding of p65 NF-κB subunit was measured using the TransFactor ELISA-based assay (Clontech).

C. A qRT-PCR assay showing the relative level of K13 expression in the BCBL1-TREx-RTA cells expressing a control vector (MSCV) or K13 as compared to the basal level of K13 expressed in the BC-1 cell line. The qRT-PCR analysis was performed in triplicate and GNB2L1was used as a normalizing control.

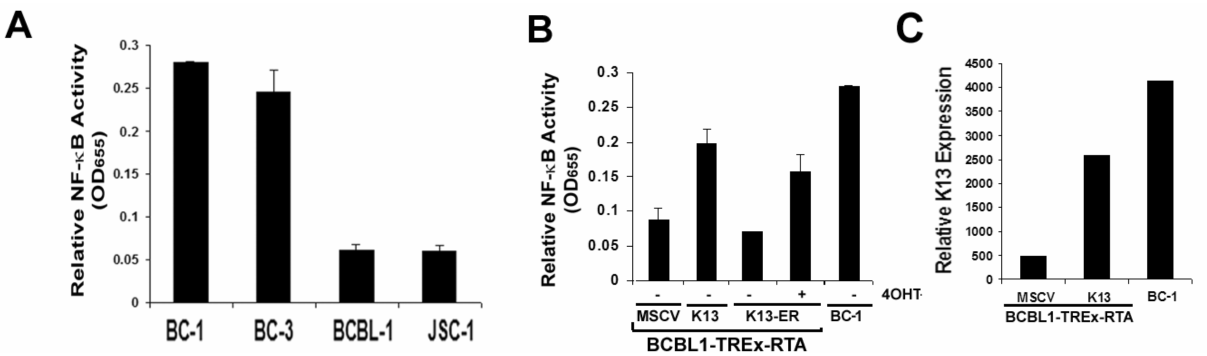

Supplement: Figure S1 — Relative level of NF-κB activity and K13 expression in PEL cells. A. Basal level of NF-κB activity in different PEL cell lines as measured by the TransFactor ELISA-based assay kit. B. A NF-κB DNA binding assay showing the NF-κB activity present in BCBL1-TREx-RTA cells expressing a control vector (MSCV), K13 or the K13-ERTAM construct (with and without 4OHT treatment) as compared to the basal level of NF-κB activity present in the BC-1 cell line. DNA binding of p65 NF-κB subunit was measured using the TransFactor ELISA-based assay (Clontech). C. A qRT-PCR assay showing the relative level of K13 expression in the BCBL1-TREx-RTA cells expressing a control vector (MSCV) or K13 as compared to the basal level of K13 expressed in the BC-1 cell line. The qRT-PCR analysis was performed in triplicate and GNB2L1was used as a normalizing control. (0.14 MB PDF) [file pone.0001067.s001.pdf]
