# Supplementary material for: K13 Blocks KSHV Lytic Replication and Deregulates vIL6 and hIL6 Expression: A Model of Lytic Replication Induced Clonal Selection in Viral Oncogenesis
Source: PLoS One. 2007 Oct 24;2(10):e1067. doi: 10.1371/journal.pone.0001067 (PMC2020437; doi:10.1371/journal.pone.0001067)

**Figure S2. K13 blocks lytic replication in JSC-1 cells.**

**A.** Expression of K13-ER$^{TAM}$ in BCBL1-TREx-RTA cells as determined by immunoblotting with a Flag antibody.

**B.** Treatment with 4-OHT induces NF-κB DNA-binding in JSC-1 cells expressing the K13-ER$^{TAM}$ fusion protein. DNA binding of p65 NF-κB subunit was measured using the TransFactor ELISA-based assay (Clontech).

**C.** K13 blocks TPA-induced ORF59 expression but fails to block vIL6 induction in JSC-1 cells. JSC-1-K13-ER$^{TAM}$ cells were left untreated or treated with 4OHT (20nM) for 24 h and then induced with TPA (20 ng/ml) for 96 h. Expression of ORF59 and vIL6 was detected by indirect immunofluorescence analysis. Nuclei were counterstained with Hoechst 33342.

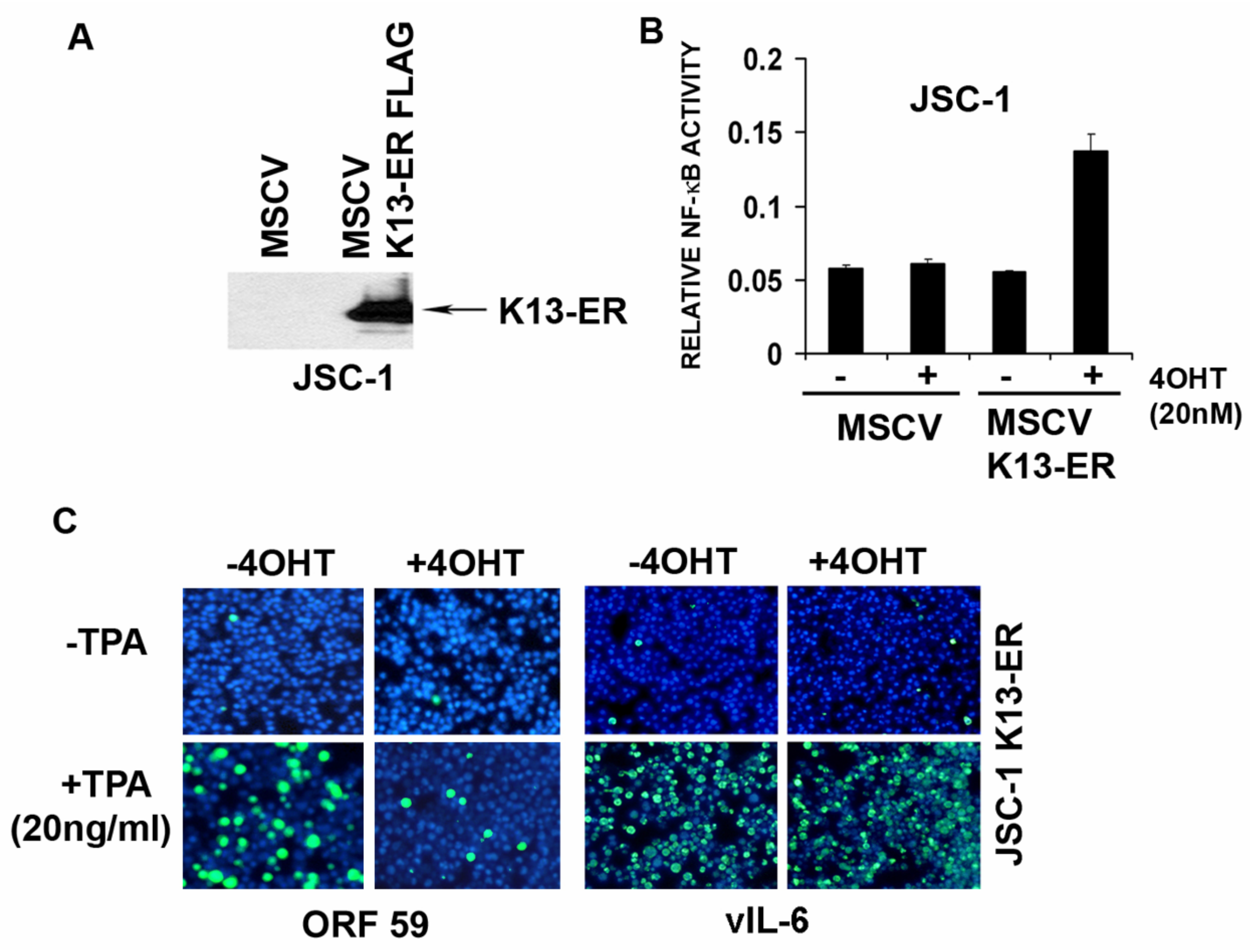

Supplement: Figure S2 — K13 blocks lytic replication in JSC-1 cells. A. Expression of K13-ERTAM in BCBL1-TREx-RTA cells as determined by immunoblotting with a Flag antibody. B. Treatment with 4-OHT induces NF-κB DNA-binding in JSC-1 cells expressing the K13-ERTAM fusion protein. DNA binding of p65 NF-κB subunit was measured using the TransFactor ELISA-based assay (Clontech). C. K13 blocks TPA-induced ORF59 expression but fails to block vIL6 induction in JSC-1 cells. JSC-1-K13-ERTAM cells were left untreated or treated with 4OHT (20 nM) for 24 h and then induced with TPA (20 ng/ml) for 96 h. Expression of ORF59 and vIL6 was detected by indirect immunofluorescence analysis. Nuclei were counterstained with Hoechst 33342. (0.72 MB PDF) [file pone.0001067.s002.pdf]
