# Supplementary material for: K13 Blocks KSHV Lytic Replication and Deregulates vIL6 and hIL6 Expression: A Model of Lytic Replication Induced Clonal Selection in Viral Oncogenesis
Source: PLoS One. 2007 Oct 24;2(10):e1067. doi: 10.1371/journal.pone.0001067 (PMC2020437; doi:10.1371/journal.pone.0001067)
Supplement: Table S1 — Sequence of siRNA oligonucleotides. (0.01 MB PDF) [file pone.0001067.s003.pdf]

Supplementary Table 1. Sequence of siRNA oligonucleotides

Name	Sequence
K13 siRNA	5'-AACGUGUUCAUACCUCAACCC-3'
Control siRNA	5'-AAGCGCGCUUUGUAGGAUUCG-3'
