# Supplementary material for: K13 Blocks KSHV Lytic Replication and Deregulates vIL6 and hIL6 Expression: A Model of Lytic Replication Induced Clonal Selection in Viral Oncogenesis
Source: PLoS One. 2007 Oct 24;2(10):e1067. doi: 10.1371/journal.pone.0001067 (PMC2020437; doi:10.1371/journal.pone.0001067)
Supplement: Table S2 — Sequence of primers used for RT-PCR and qRT-PCR analyses. (0.01 MB PDF) [file pone.0001067.s004.pdf]

Supplementary Table 2: Sequence of primers used for RT-PCR and qRT-PCR analyses.

Gene Name	Forward Primer	Reverse Primer
K1	AAACAACGTGACTCAAACAAAACA	TCTTCCGTGCACAAATCGTG
K2	ACCCTTGCAGATGCCGG	GGATGCTATGGGTGATCGATG
K4	TTGTCCGGTCTATGCCAGG	CTGCCTTGCTTTGTTTGCAA
K8.1	AAAGCGTCCAGGCCACCACAGA	GGCAGAAAATGGCACACGGTTAC
K9/vIRF-1	CGGCATAGCTGTGCTTACCA	CATTGTCCCGCAACCAGACT
K10	CCCAACAGGCCAGCTACATAA	CTTCGTGGAACTCTGAGACGC
K11	ATCCGAGTCATATTCAGGCGA	AATCGAGAACCTGAAGGGTCC
K13	GGATGCCCTAATGTCAATGC	GGCGATAGTGTTGGAGTGT
ORF50	CACAAAAATGGCGCAAGATGA	TGGTAGAGTTGGGCCTTCAGTT
GNB2L	GAGTGTGGCCTTCTCCTCTG	GCTTGCAGTTAGCCAGGTTC
